# A Georgia Wonder

**Published:** 1888-08

**Authors:** 


### A GEORGIA WONDER.

The Memphis Avalanch gives some interesting particulars of the manifestations, a mysterious force in the presence of Miss Dixie Haygood, (now Mrs. Embury), which it describes as supernatural. How long will it be ere we shall be driven to recognize that none of the happenings of our world are supernatural ; that nothing here can exist in opposition to natural law, that the phenomena which take place in the presence of this peculiarly gifted woman are in as strict accordance with that law as the commonest occurrences which, from their frequency, excite no wonder. It seems that the Avalanche reporter was a "Skeptic on all such matters, and did not believe in ghosts, spirits, or other supernatural phenomena, and it was his determination, as also that of several of the other gentlemen present, to detect, if possible, any trickery or legerdemain that might be attempted.

"Miss Haygood is of small stature, compact mold, and apparently muscular beyond most women, and weighs about 104 pounds. She has brown hair, blue or violet eyes, and a pleasant expression of countenance, but an air also of firmness and decision of character. The first two tests, viz., drawing a gentleman easily around the room by applying her hand to a chair or an umbrella held by him, or the third test of holding a billiard cue in her open palms at an angle of about 45 degrees with such firmness that a strong man or even two strong men, could not force the point to the floor, were certainly remarkable in a woman so small, but might have been accomplished by a woman of very great physical strength. Therefore, the reporter felt disappointed to some extent, and argued with his companions that it was either a matter of sleight or else a remarkable development of muscular power."

"But the next tests were different. One of the gentlemen present was requested to lift the lady by her elbows, held taut at the waist. This he did, but when told to try it again utterly failed to raise her an inch. Two strong men then raised her by holding her elbows on each side with the greatest ease, but on attempting it again they could not raise her weight a particle from the floor. This was a poser and was tried several times, the party offered many explanations, but none at all satisfactory. The fact remained unexplained that she could at will allow herself to be lifted as other people, and immediately thereafter bring into play such a force without apparent effort, that two strong men could not move her 100 pounds of weight, try they ever so hard."

"The next test was still more inexplicible. A chair was brought—an ordinary dining room chair—and a gentleman weighing about 130 pounds was placed on it. Astride of his knees another gentleman of about 145 pounds weight was placed, and between the two a third gentleman of at least 150 pounds was seated. All raised their feet from the floor as the chair was tilted back and held balanced. The little lady then approached the back of the chair and placed her open palms along the arms or staffs supporting the chair's back."

"Observing the reporter narrowly watching her, she requested him to place his hands between her hand and the chair to prove that no pressure was exerted. This was done, and only the lady's thumbs touched the back of the chair. Then, without an effort or the centraction of a muscle, or the slightest pressure on the reporter's hand, the chair and its living freight was raised about 14 inches from the floor. The weight, in cluding the chair, was at least 450 pounds, and John L. Sullivan could not have performed the feat so easily accomplished by the little woman."

"This test was convincing, but the next was no less so. Miss Haygood stood on one foot and holding a billiard cue horizontally before her at half arm's length, three strong men essayed in vain to push her, by throwing their combined weight against the cue, from her balance on one foot or press her arms back to her chest. The reporter offers no explanations of these strange exhibitions of hidden force. He saw the shapely, womanly hands lift the three great strapping fellows from the floor by simply touching with open palms his own hands placed on the chair back, and he saw the dainty No. two-and-a-half boot standing unmoved on the floor with the combined weight of the three Memphis gentlemen thrown powerfully and persistently against the small figure of its owner. Explain it who can."

None can explain it upon any theory outside of what is known as mediumship and manifestations of spirit control. To the students of the occult science the explanation is simple enough.

